# Epidemiology and Clinical Outcomes of COVID-19 Patients in Northwestern China Who Had a History of Exposure in Wuhan City: Departure Time-Originated Pinpoint Surveillance

**DOI:** 10.3389/fmed.2021.582299

**Published:** 2021-05-28

**Authors:** Qingqing Zhang, Jianfei Zhu, Chenghui Jia, Shuonan Xu, Tao Jiang, Shengyu Wang

**Affiliations:** ^1^Department of Pulmonary and Critical Care Medicine, The First Affiliated Hospital of Xi'an Medical University, Xi'an, China; ^2^Department of Thoracic Surgery, Tangdu Hospital, Air Force Military Medical University (Fourth Military Medical University), Xi'an, China; ^3^Department of Thoracic Surgery, Shaanxi Provincial People's Hospital, Xi'an, China; ^4^Department of Cardiothoracic Surgery, The First Affiliated Hospital of Xi'an Medical University, Xi'an, China

**Keywords:** outcomes, exposure history, departure time, epidemiological terms, COVID-19

## Abstract

**Background:** Most COVID-19 patients cannot provide a clear exposure time; therefore, this study was designed to predict the progression of COVID-19 by using the definite departure time from Wuhan.

**Methods:** In this retrospective study, all cases were selected from Northwestern China, which has the lowest population density. As our study endpoints, the incubation period was defined as the date of departure from Wuhan City to the date of symptom onset; we defined the confirmed time as the interval from symptom onset to positive results (samples from the respiratory tract). Both of them were estimated by fitting a Weibull distribution on the departure date and symptom onset. The differences among the variables were analyzed.

**Results:** A total of 139 patients were ultimately enrolled, and ~10.1% of patients (14 patients) had no symptoms during their disease course. We estimated the median incubation period to be 4.0 days (interquartile intervals, 2.0–8.0), and the 95th percentile of the distribution was 15.0 days. Moreover, ~5.6% of patients (7 patients) experienced symptoms 2 weeks after leaving. Furthermore, the estimation median interval from symptom onset to final diagnosis was 4.0 days (interquartile intervals, 2.0–6.0), and the 95th percentile of the distribution was 12.0 days. Finally, the median hospitalization time was 16.0 days, ranging from 3.0 to 45.0 days. Univariate analysis showed that age (*P* = 0.021) and severity status (*P* = 0.001) were correlated significantly with hospitalization time.

**Conclusions:** We provide evidence that departure time can be used to estimate the incubation and confirmed times of patients infected with COVID-19 when they leave an epidemic area.

## Introduction

From December 2019 until March 11, 2020, 3,173 Chinese people died of COVID-19 (1). Compared with the SARS breakout in 2003, COVID-19 presented the following characteristics (2–7): (1) higher infection; (2) lower lethality; (3) infections in the incubation period; (4) asymptomatic patients are also contagious; (5) multiple organ susceptibility. Thus, it is indispensable to investigate the epidemiological characteristics of COVID-19, especially for patients with exposure histories in epidemic areas.

Definite exposure time is critical for analyzing infectious diseases, especially for respiratory tract infectious diseases that were spread through short-range droplets, such as influenza. Previous studies have indicated that the main routes of transmission of COVID-19 were droplets and aerosols (8); other researchers (9) reported that healthy carriers could also transmit the virus. Wuhan city is a megalopolis of high population density in China. Due to the aforementioned factors, we could not obtain definite exposure times, meaning that it was hard to estimate the incubation period of COVID-19. Guan et al. (10), in a retrospective study enrolling 1,099 patients, reported that only 289 patients had information on their specific date of exposure. Other scholars (11) dealt with this situation by choosing the date the first reported patient presented symptoms, which is obviously not rigorous.

Since March 11, the coronavirus has spread to more than 123 countries and regions, with ~132,000 cases infected with COVID-19, as reported by WHO, spurring WHO to characterize the outbreak as a pandemic. With the global outbreak (12), all countries are facing hard work to prevent and control both domestic epidemics and imported cases from hardest hit areas; however, imported cases or suspicious ones cannot provide definite exposure times, making it hard to calculate the incubation period of patients and to establish the length of time for quarantine and medical observation.

For imported cases, the departure time can be accurately obtained in real-world studies. Therefore, this study was designed to predict the progression of COVID-19 by using definite departure times, which could be easily provided by patients, as the exposure time, and all patients experienced exposure history in Wuhan City. To reduce the possibility of secondary exposure caused by population density and mobility, we selected patients who received treatment at designated hospitals in Northwestern China, the area with the lowest population density.

## Materials and Methods

### Study Design

This study was designed to analyze the epidemiological characteristics and clinical outcomes of patients from Northwestern China diagnosed with novel coronavirus pneumonia (NCP) who had history of exposure in Wuhan City. The degree of severity, diagnostic criteria, choice of treatment mode, and discharge standard refer to the 7th edition of the National New Coronavirus Pneumonia Diagnosis and Treatment Program. To reduce the possibility of secondary exposure caused by population density and mobility, we selected the patients who received treatment at designated hospitals distributed in Northwestern China, the area with the lowest population density. This area includes four autonomous regions and three provinces. All cases enrolled in this study fulfilled the following criteria: (1) had exposure history in Wuhan City; (2) without a definite exposure date; (3) COVID-19 virus nucleic acid results were positive; (4) no direct contact with confirmed or suspected patients after leaving Wuhan City; (5) symptom appearance after leaving Wuhan city; (6) treated at a designated hospital; (7) with definite disease outcome (death or discharge). The last follow-up time was March 11, 2020. This study was approved by the ethics committee of the First Affiliated Hospital of Xi'an Medical University (No. XYYFY2020LSK-026). Written informed consent was waived due to the nature of open-access data, and it was approved by the First Affiliated Human Research Ethics Committee of Xi'an Medical University. All procedures followed were in accordance with the Declaration of Helsinki.

### Setting

These areas are located in Northwestern China, far from Wuhan City. They include four autonomous regions (Inner Mongolia Autonomous Region, Tibet Autonomous Region, Xinjiang Uygur Autonomous Region, and Ningxia Hui Autonomous Region) and three provinces (Gansu Province, Qinghai Province, and Shaanxi Province) (Table 1). These regions and provinces account for 57.5% of the total territory of China; however, the population density of the area is only 23.8 persons/km^2^, which is lower than the national average population density (145.4 persons/km^2^).

**Table 1:** Association between clinical characteristics and severity status of patients diagnosed with COVID-19.

**Characteristics**		**Severe/critical cases**	***P***
**Age (median: 36years, rang from 1 to 71)**	***N*** **(139)**	***N*** **(%)**	
Gender			0.051
Male	92	8 (100%)	
Female	47	0 (0)	
Religion			0.645
Shaanxi province	67	5 (62.5%)	
Qinghai province	14	1 (12.5%)	
Tibet autonomous region	1	0 (0)	
Xinjiang Uygur autonomous region	1	0 (0)	
Ningxia Hui autonomous region	12	0 (0)	
Gansu province	23	0 (0)	
Inner Mongolia autonomous region	21	2 (25.0%)	
Age^a^			0.719
<36	68	3 (37.5%)	
≥36	71	5 (62.5)	
Exposure history			0.049
Resident	98	3 (37.5%)	
Traveler	41	5 (62.5%)	
Symptom			0.669
Fever	94	7 (87.5%)	
Cough	12	1 (12.5%)	
Diarrhea	3	0 (0)	
Other symptom	16	0 (0)	
Without symptom	14	0 (0)	

a *Age: median age*.

### Data Collection

We obtained the data from the news and press releases reported by the provincial and local municipal Center for Disease Control and Prevention (CDC) or the Health Commission. The date of leaving Wuhan City, the date of symptom onset, the date of diagnosis, the date of discharge, age, gender, and other patient-related data were extracted from the news and press releases. Four reviewers (JZ, CJ, QZ, and SX) collected the data independently, and data were verified with the National Health Commission and Chinese CDC. Major disagreements between these four doctors were checked by all doctors together.

### Definition

**Figure 1:**
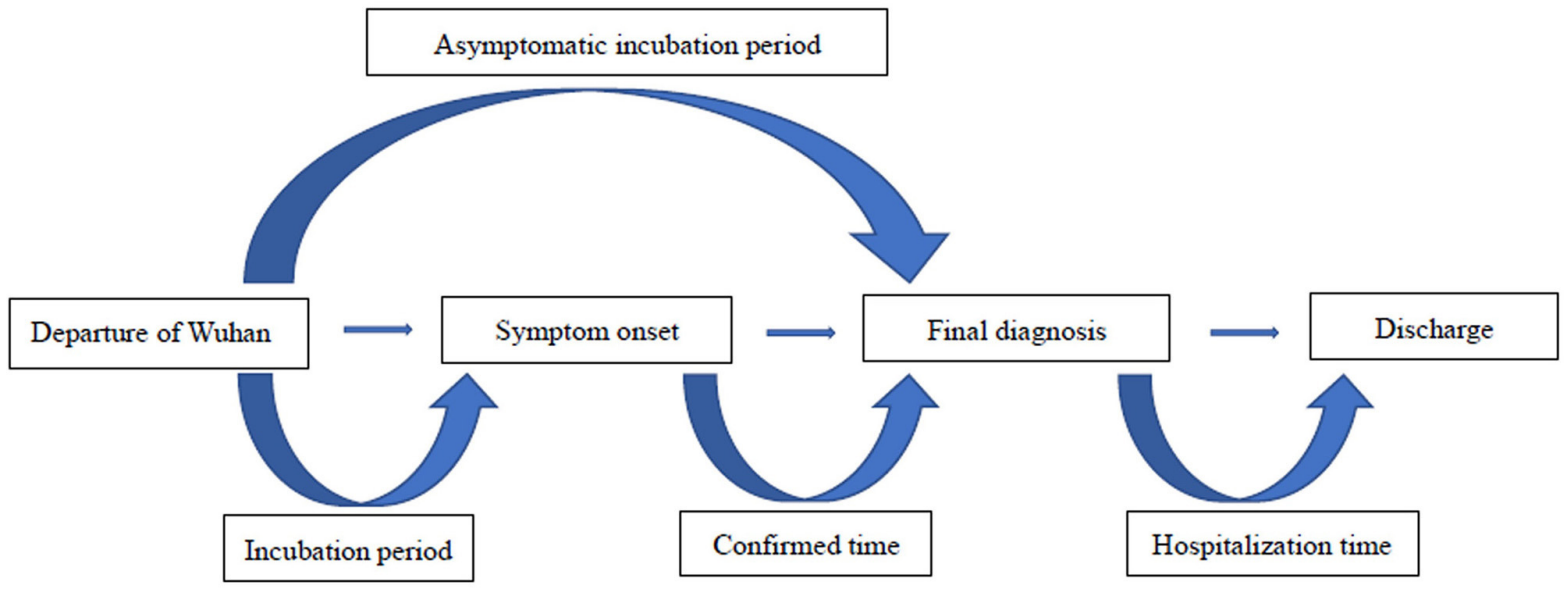
Definitions of the main endpoints of this study.

The residents were confirmed to have stayed in Wuhan City more than 2 weeks during the outbreak. The incubation period was defined as the date of departure from Wuhan City to the date of symptom onset or the date of final diagnostic time (asymptomatic patient); we defined the confirmed time as the interval from symptom onset to positive results (samples from the respiratory tract). Hospitalization time is recognized as from final diagnosis to date of discharge or death (Figure 1).

### Statistical Analysis

Categorical variables are summarized as numbers and percentages. We estimated the incubation period and the confirmed time by fitting a Weibull distribution on the dates of departure and symptom onset. The relationship between severity status of COVID-19 and clinical characters was analyzed by using a χ^2^-test. Normal distribution and homogeneity of variances were tested, *T*-test and variance analysis were performed to compare the difference among the variables, and the Mann–Whitney U-test and Kruskal–Wallis H-test were applied when the cases did not fit the normal data distribution. Bilateral *P* ≤ 0.05 was considered statistically significant. All analyses were performed using SPSS software (version 22.0), and Weibull fitting distribution was estimated by MATLAB 18.0.

## Results

### Clinical Characteristics and Severity Status

**Figure 2:**
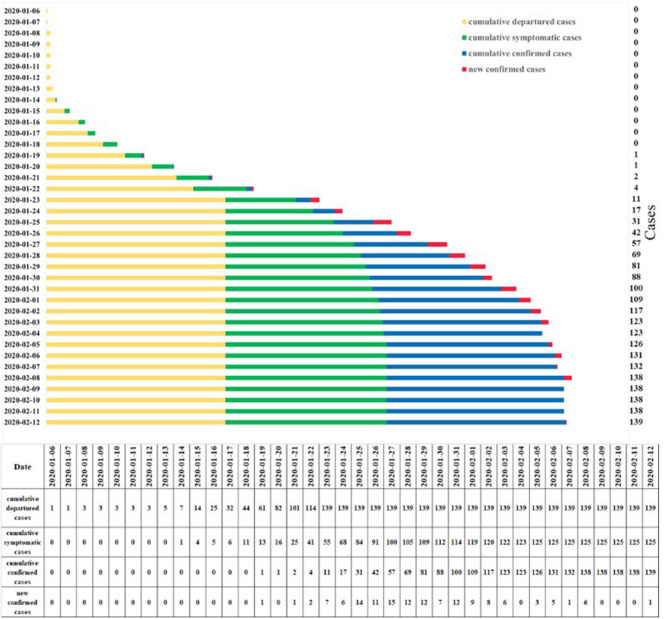
Time distribution of all patients after they left Wuhan City.

As of March 11, 2020, a total of 139 patients diagnosed with COVID-19 were enrolled in this study. All patients were from Northwestern China and were verified to have an exposure history in Wuhan City. Figure 2 shows the time distribution of all patients; the earliest and latest times to leave Wuhan City were January 6, 2020 and January 23, 2020, respectively. Only one patient was provided by both Tibet Autonomous Region and Xinjiang Uygur Autonomous Region. The largest number of patients (67 patients) was provided by Shaanxi Province, accounting for 48.2% (Table 1).

Of the 139 patients, 92 patients were male and 47 patients were female; the median age of the patients was 36 years (range: 1–77 years). Approximately 70.5% of patients (98 patients) were residents, and 41 patients were travelers. A total of 94 patients had the common symptom of fever (75.2%), ~2.4% of patients (3 patients) presented diarrhea, 12 patients presented only cough (9.6%), and 12.8% of patients presented other symptoms. According to severity status, ~94.2% of patients were categorized as general, 4 patients (2.9%) as severe, and 4 patients (2.9%) as critical; severe and critical patients were analyzed together. Patients with short-term exposure (travelers) were more likely to develop severe or critical status than those with long-term exposure (residents) (62.5 vs. 37.5%, *P* = 0.049). Of the eight severe or critical patients, all of them were male (*P* = 0.051) (Table 1).

### Epidemiological Characteristics

**Figure 3:**
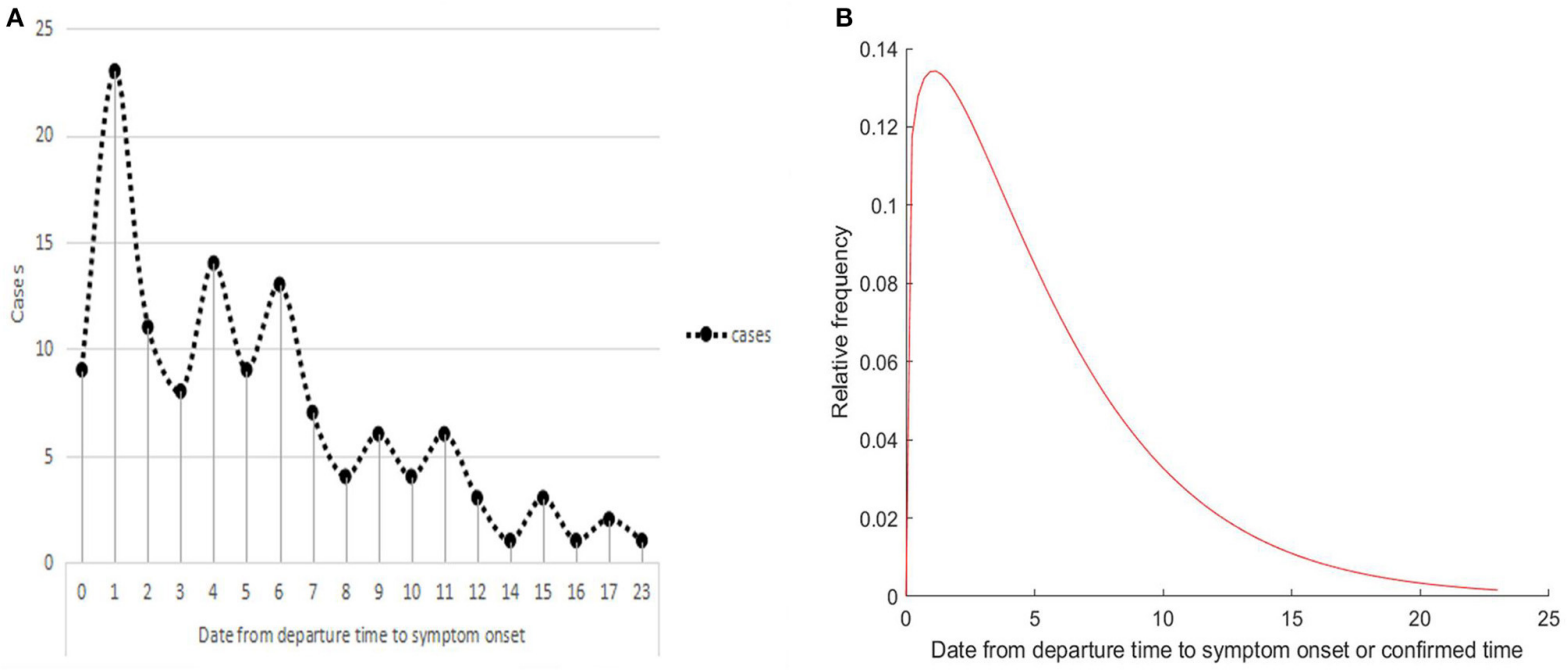
The incubation period of COVID-19. **(A)** Frequency distribution of incubation periods. **(B)** Estimation of incubation period by fitting a Weibull distribution.

Interestingly, ~10.1% of patients (14 patients) were healthy carriers, without any symptoms during their disease course. For asymptomatic patients, ~57.1% of patients (8/14) were determined to be positive for nucleic acid of COVID-19 virus within 5.0 days of leaving Wuhan City. For symptomatic patients, two patients presented onset of symptoms after they were confirmed (1.6%), and one of them presented symptoms after 9 days. Most patients (75.2%) had onset of symptoms within a week (94/125), nearly 5.6% of patients (7 patients) experienced symptoms 2 weeks after leaving, and one patient developed symptoms after 23.0 days. In addition, the peak time of symptom onset emerging after they left the epicenter was on the first day (23 patients) (Figure 3A). All 139 patients diagnosed with COVID-19 were estimated by fitting the Weibull distribution; the median incubation period was 4.0 days (interquartile range, 2.0–8.0), and the 95th percentile of the distribution was 15.0 days (Figure 3B).

**Figure 4:**
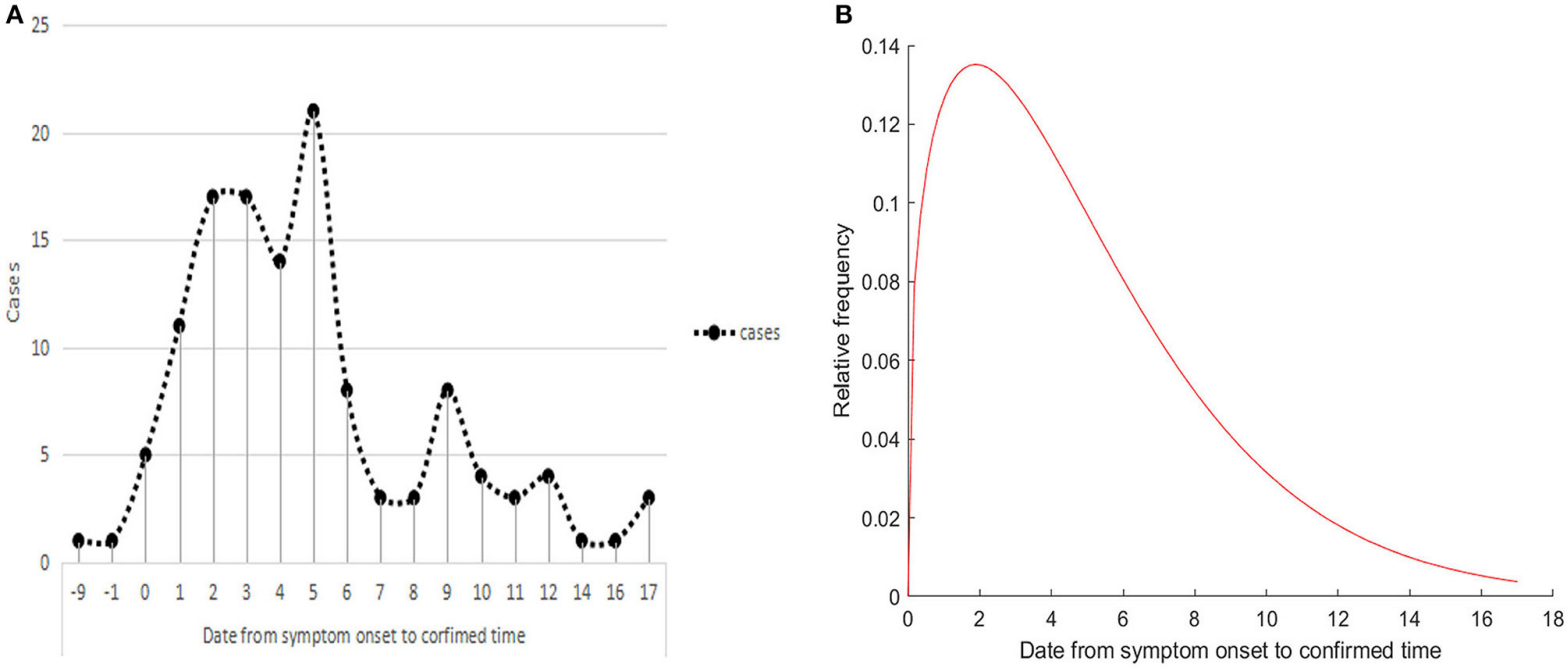
The confirmed time of COVID-19. **(A)** Frequency distribution of confirmed times. **(B)** Estimation of the confirmed time by fitting a Weibull distribution.

Our study purpose was to address the issue of the optimal detection opportunity after symptom onset. The median confirmed time was 4.0 days (−9 to 17.0 days). Figure 4A shows that the peak of confirmed time was on the 5th day after symptom onset, and ~68.0% of patients presented positive results within 5 days of symptom onset. For 125 symptomatic patients, the median interval from symptom onset to final diagnosis was 4.0 days (interquartile range, 2.0–6.0), and the 95th percentile of the distribution was 12.0 days (Figure 4B).

The time of positive detection of older patients (≥36 years) was later than that of younger patients (<36 years) after symptom onset (5.0 vs. 4.0 days, *P* = 0.028); in addition, male patients had later positive results detected (5.0 days, interquartile range: 3.0–7.0) compared with female patients (3.0 days, interquartile range: 2.0–5.0, *P* = 0.051).

### Clinical Outcomes

Since March 11, 2020, all patients have been discharged; they all recovered. The median hospitalization time was 17.0 days, ranging from 3.0 to 45.0 days. Further analyses showed that the following variables were correlated significantly with hospitalization time: age (*P* = 0.021) and severity status (*P* = 0.001) (Table 2).

**Table 2:** The epidemiological characteristics and clinical outcomes of patients diagnosed with COVID-19.

**Variable**	**Symptom onset to confirmed time**	**IQI**^**b**^	***P***	**Hospitalization time**	**IQI**^**b**^	***P***
**In total**						
Gender			0.051			0.085
Male	5	(3, 7)		16	(11, 20)	
Female	3	(2, 5)		18	(13, 21)	
Age^a^			0.028			0.021
<36	4	(2, 5)		15.5	(12, 19)	
≥36	5	(2.5, 9)		18	(12, 22)	
Exposure history			0.145			0.881
Resident	4	(2, 6)		16	(12, 21)	
Traveler	5	(3, 9)		18	(11.5, 20)	
Symptom			0.801			0.127
Fever	4	(2, 6)		17	(13, 21)	
Cough	4	(2, 5)		20	(10.25, 27.75)	
Diarrhea	4	(2.5, 6.5)		12	(9.5, 14.75)	
Other symptom	4	(3, 9)		16	(11.25, 19.75)	
Without symptom				13.5	(9, 17.75)	
Severity status			0.414			0.001
General	4	(2, 6)		16	(12, 20)	
Severe	8	(1.5, 10.75)		23.5	(19.75, 31)	
Critical	2.5	(−0.25, 9)		34	(22.25, 44.25)	

a *Age: median age*.

b *IQI: interquartile intervals*.

## Discussion

This retrospective study estimated the incubation period for COVID-19 by using the departure time from Wuhan City as the exposure time because in real-world studies, it is difficult to determine the definite exposure time. We collected the patients from a lower-population-density area in Northwestern China to reduce the possibility of secondary exposure; thus, our data had practical guidance value for preventing and controlling COVID-19, especially for patients or persons with suspicion of infection from the epicenter. Our data showed that the median incubation period of COVID-19 was 4.0 days after leaving the epidemic area.

Our results were basically consistent with other studies (2, 11, 13), although the definition of exposure time was different. A retrospective study from China showed that the mean incubation period of COVID-19 was 4.0 days (95% CI, 2.0–7.0); however, while a total of 1,099 patients were enrolled in this study, only 291 had a clear exposure time. All patients were Chinese. Lauer et al. (11) estimated the incubation period of COVID-19 in his study: 181 patients from 24 countries or regions were analyzed, and the median incubation period was estimated to be 5.1 days (95% CI, 4.5–5.8 days). Other related research reported longer incubation periods than ours. An epidemiological surveillance of early confirmed COVID-19 patients in Shanghai (14) showed that the mean incubation period was 6.4 days (95% CI 5.3–7.6), and the 95th percentile was 13.1 days. Data from Henan Province (15) of China estimated that the average latency of 483 patients was 7.4 days, and over 92.0% of patients had an incubation period of <2 weeks. We also found that ~75.2% patients developed symptoms within 1 week after leaving the epidemic area (Wuhan City). It was noteworthy that 5.6% of patients presented symptoms 2 weeks later, while one patient's symptoms appeared 23.0 days later.

Interestingly, ~10.1% of (14 patients) did not present any clinical symptoms in their disease course. It is controversial whether asymptomatic patients are contagious. A study from Chinese researchers showed (16) that at least 59.0% of infection cases in Wuhan City might had not been identified, which may include those who are asymptomatic or who have mild symptoms. The MedRxiv platform published research from American scholars (17) that suggested that there may be a small percentage of infected individuals who are asymptomatic and can transmit the COVID-19 virus. An asymptomatic carrier from Henan Province of China transmitted COVID-19 to her five family members; her incubation period was as high as 19.0 days (9). Moreover, some research has indicated that there was no difference in the virus load between asymptomatic patients and symptomatic patients (18, 19). However, other studies showed the opposite result (20). In our study, the results of COVID-19 nucleic acid testing were positive, which indirectly proved that asymptomatic patients are contagious compared with symptomatic patients; thus, we should pay more attention to asymptomatic patients.

Further studies have shown that most asymptomatic patients are categorized as general (5, 21), meaning that these patients have better outcomes. However, the next generation of patients who become infected by these asymptomatic individuals might have worse outcomes (22); the specific mechanism of viral pathogenesis is unknown. In our study, for asymptomatic patients, ~68.0% patients were detected as positive for COVID-19 viral nucleic acid within 5.0 days of leaving Wuhan City due to the early detection of suspicious populations by the government. Shao and Shan (23) constructed a SEIR model and suggested that medical examinations should be performed on exposed or potentially exposed individuals.

In concert with recent studies, fever was the most common symptom (75.2%) diagnosed in this study, which was consistent with the results of the meta-analysis by Sun et al. (24). For patients diagnosed with COVID-19, laboratory test results that did not match clinical symptoms were found in these studies (25–27). Two patients experienced symptoms after they were diagnosed (1.6%), and one presented symptoms after 9 days; thus, all suspicious populations should be observed dynamically.

This study also focused on the optimal time to detect COVID-19 nucleic acids. We found that the median confirmation time was 4.0 days. A recent retrospective study from Tongji Hospital (28) proved that the median time from symptom onset to confirmation was 16.0 days, longer than our data. They also found that ~30.0% of these patients had positive results for the third time; meanwhile, they found that positive results were detected later in older patients (≥65 years) (18.0 vs. 14.0 days, *P* < 0.001), consistent with our data. Our results also indicated that most positive results appeared on the fifth day after symptom onset. Similar to our results, the clinical sensitivity of RT-PCR on swabs taken on the first day to the fifth day after symptom onset was 100% (29).

We also analyzed the factors that affect the patient's hospitalization time. In our study, the median hospitalization time was 17.0 days, ranging from 3.0 to 45.0 days. Further analyses showed that the following variables were correlated significantly with hospitalization time: age (*P* = 0.021) and severity status (*P* = 0.001), suggesting that age and severity status might been the prognostic factors for patients with COVID-19. A study from China had confirmed that older age associated with patient's in-hospital death (OR: 1.10, 95% CI: 1.03–1.17, *P* = 0.0043) (30). The possible explanation was that age and the severity of pneumonia will increase the occurrence of cardiac events after pneumonia, leading to a poor prognosis for patients (31). Therefore, we should pay more attention to these patients.

The limitations of this study should be noted. First, the laboratory results had not been analyzed because data sources from news and press releases were reported by Provincial and Autonomous CDCs. Second, because this was a retrospective study, further analysis was limited due to the small sample size of this study. Finally, although our results were broadly consistent with the related research, patients with exact exposure times should be included in future analyses for comparison.

In conclusion, we provide evidence that departure time can be used to estimate the incubation and confirmed times of patients infected with COVID-19 when they leave an epidemic area. The median of the incubation period was 4 days, and 5.6% of patients experienced symptoms 2 weeks after leaving. The longest time was 23.0 days from the date of departure, suggesting that the length of time for quarantine and medical observation, now recognized as 2 weeks, might not be sufficient. Moreover, most patients were detected to be positive for viral nucleic acid within 5.0 days of when symptoms appeared. Finally, healthy carriers should be given more attention.

## Data Availability Statement

The original contributions presented in the study are included in the article/supplementary material, further inquiries can be directed to the corresponding author/s.

## Ethics Statement

The studies involving human participants were reviewed and approved by the ethics committee of the First Affiliated Hospital of Xi'an Medical University (No. XYYFY2020LSK-026). The patients/participants provided their written informed consent to participate in this study.

## Author Contributions

TJ and SW participated in study design and study conception. QZ, JZ, and CJ performed data analysis and drafted the manuscript. QZ, JZ, CJ, and SX recruited patients. All authors provided critical review of the manuscript and approved the final draft for publication.

## Conflict of Interest

The authors declare that the research was conducted in the absence of any commercial or financial relationships that could be construed as a potential conflict of interest.
